# Supplementary material for: Peptide inhibition of neutrophil-mediated injury after in vivo challenge with supernatant of Pseudomonas aeruginosa and immune-complexes
Source: PLoS One. 2021 Jul 9;16(7):e0254353. doi: 10.1371/journal.pone.0254353 (PMC8270186; doi:10.1371/journal.pone.0254353)
Supplement: S1 Fig — Panel A and B show Intraperitoneal MPO dose ranging experiments Increasing amounts of purified MPO was injected IP and after 1 hour animals underwent phlebotomy, euthanasia and peritoneal wash. A) Peritoneal wash supernatant oxidation of TMB (n = 4). Data are means of independent animals ±SEM. B) Peritoneal wash supernatant free DNA measured via PicoGreen assay (n = 4). Data are means of independent animals ±SEM. Panel C and D show Intraperitoneal MPO time course experiments Purified MPO (0.1 mg) was injected IP and at increasing intervals animals underwent phlebotomy, euthanasia and peritoneal wash. C) Peritoneal wash supernatant oxidation of TMB (n = 4). Data are means of independent animals ±SEM. D) Peritoneal wash supernatant free DNA measured via PicoGreen assay (n = 4). Data are means of independent animals ±SEM. (PDF) [file pone.0254353.s001.pdf]

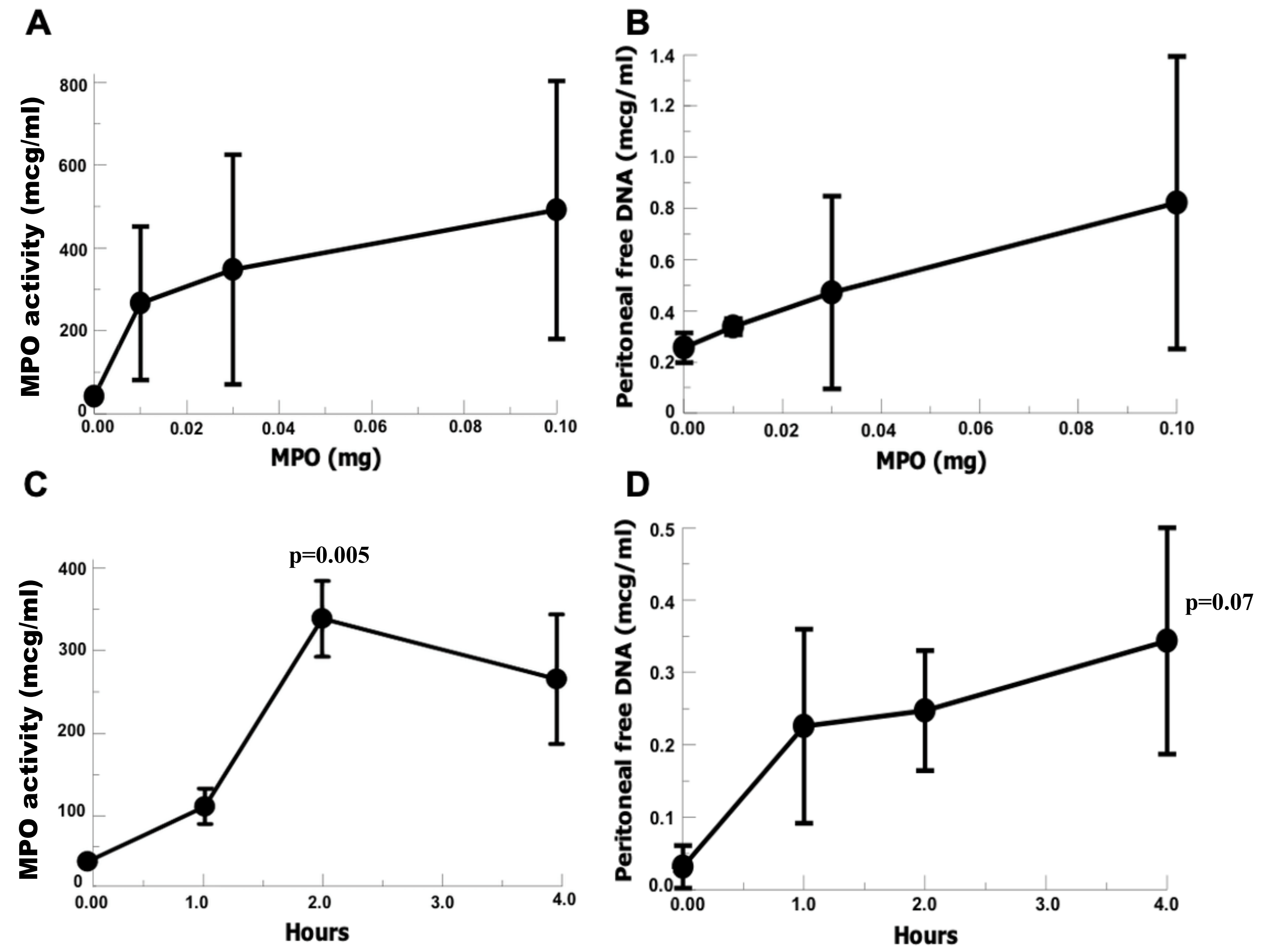

S1 Fig. Intraperitoneal MPO dose ranging and time course experiments

Panel A and B show Intraperitoneal MPO dose ranging experiments

Increasing amounts of purified MPO was injected IP and after 1 hour animals underwent phlebotomy, euthanasia and peritoneal wash. A) Peritoneal wash supernatant oxidation of TMB (n = 4). Data are means of independent animals ±SEM. B) Peritoneal wash supernatant free DNA measured via PicoGreen assay (n = 4). Data are means of independent animals ±SEM.

Panel C and D show Intraperitoneal MPO time course experiments

Purified MPO (0.1 mg) was injected IP and at increasing intervals animals underwent phlebotomy, euthanasia and peritoneal wash. C) Peritoneal wash supernatant oxidation of TMB (n = 4). Data are means of independent animals ±SEM. D) Peritoneal wash supernatant free DNA measured via PicoGreen assay (n = 4). Data are means of independent animals ±SEM.
