# Supplementary material for: Peptide inhibition of neutrophil-mediated injury after in vivo challenge with supernatant of Pseudomonas aeruginosa and immune-complexes
Source: PLoS One. 2021 Jul 9;16(7):e0254353. doi: 10.1371/journal.pone.0254353 (PMC8270186; doi:10.1371/journal.pone.0254353)
Supplement: S2 Fig — Rats received IP injection with thioglycolate to recruit neutrophils and then IP injection with Ps.a supe or saline. After euthanasia, peritoneal lavage was performed, peritoneal fluid recovered, and Wright stain was performed. A) Saline injection rat peritoneal fluid cytospin stain shows neutrophils B) Ps.a supe injection rat peritoneal fluid cytospin shows neutrophils. Representative images are shown for each group. (PDF) [file pone.0254353.s002.pdf]

A B

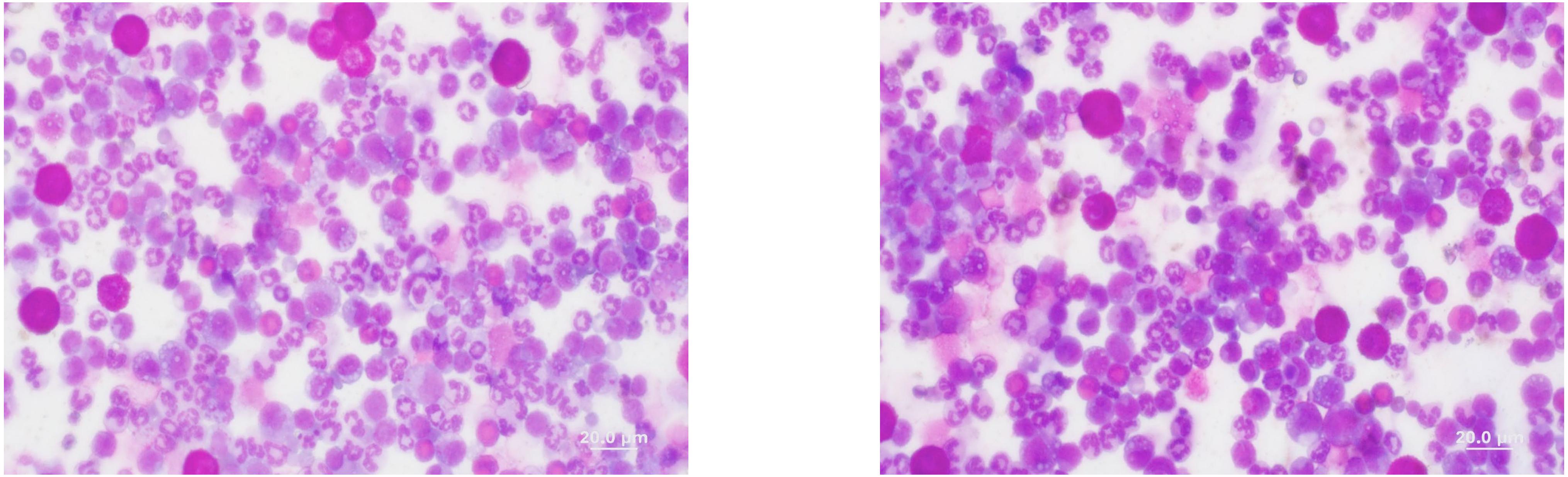

S2 Fig. Neutrophilia in *P. aeruginosa* supernatant (Ps.a supe) induced peritonitis. Rats received IP injection with thioglycolate to recruit neutrophils and then IP injection with Ps.a supe or saline. After euthanasia, peritoneal lavage was performed, peritoneal fluid recovered, and Wright stain was performed. A) Saline injection rat peritoneal fluid cytospin stain shows neutrophils B) Ps.a supe injection rat peritoneal fluid cytospin shows neutrophils. Representative images are shown for each group.
