# Supplementary material for: Peptide inhibition of neutrophil-mediated injury after in vivo challenge with supernatant of Pseudomonas aeruginosa and immune-complexes
Source: PLoS One. 2021 Jul 9;16(7):e0254353. doi: 10.1371/journal.pone.0254353 (PMC8270186; doi:10.1371/journal.pone.0254353)
Supplement: S3 Fig — Representative images show Pseudomonas-initiated neutrophil extracellular trap (NET) formation in peritonitis fluid. NET formation assayed by neutrophil elastase (NE) probed with anti-neutrophil elastase (αNE) antibody (A), fluorescence microscopy for DNA (DAPI) (B), histone H3 probed with anti-histone H3 (αhistone) antibody (C). Panel D shows the 3 stains (A,B and C) superimposed demonstrating co-localization confirming that these are NETs. (PDF) [file pone.0254353.s003.pdf]

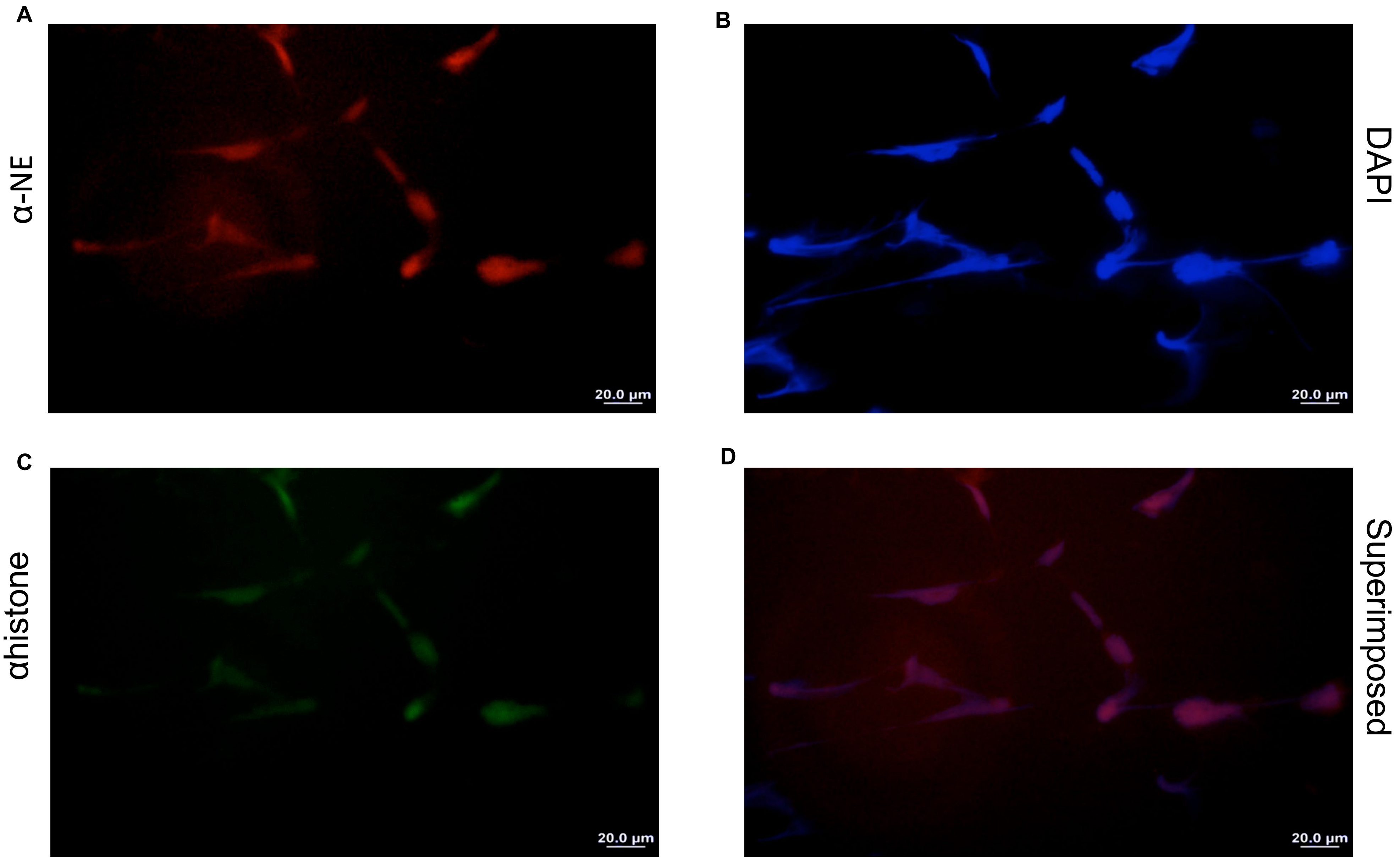

S3 Fig. *P. aeruginosa* supernatant injection induces NETosis in peritoneal wash slide @60x

Representative images show Pseudomonas-initiated neutrophil extracellular trap (NET) formation in peritonitis fluid. NET formation assayed by neutrophil elastase (NE) probed with anti-neutrophil elastase (αNE) antibody (A), fluorescence microscopy for DNA (DAPI) (B) , histone H3 probed with anti-histone H3 (αhistone) antibody (C). Panel D shows the 3 stains (A,B and C) superimposed demonstrating co-localization confirming that these are NETs.
